# Supplementary material for: LINE-1 retrotransposon expression in cancerous, epithelial and neuronal cells revealed by 5′ single-cell RNA-Seq
Source: Nucleic Acids Res. 2023 Feb 6;51(5):2033–45. doi: 10.1093/nar/gkad049 (PMC10018344; doi:10.1093/nar/gkad049)
Supplement: gkad049_Supplemental_Files [file gkad049_supplemental_files.zip › scLINE1_supplemental_figures.pdf]

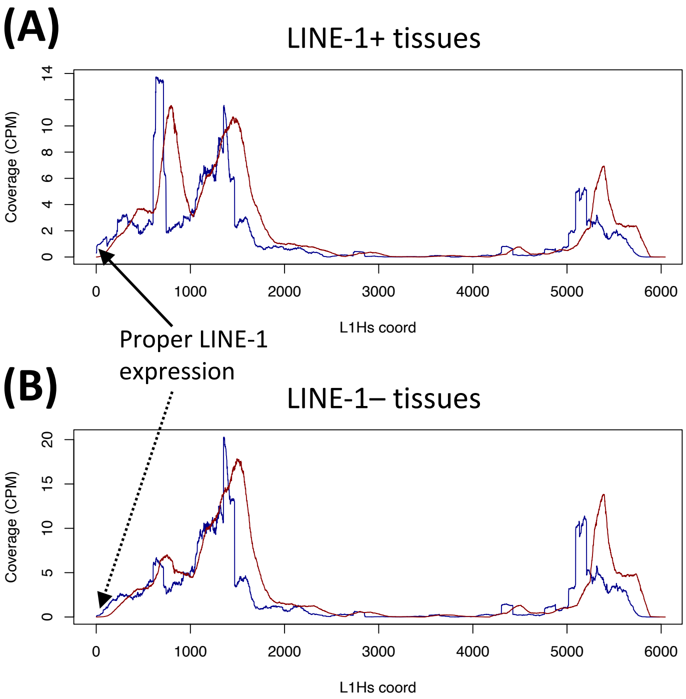

Figure S1. Coverage of L1Hs aligning reads in (A) normal LINE-1+ (bladder, esophagus, rectum, skin, small intestine, stomach, trachea) and (B) LINE-1– tissues (blood, bile duct, heart, liver, lymph node, marrow, muscle, spleen). A small peak of read 1s starting at the 5' end of LINE-1 can be seen in the LINE-1+, but not the LINE-1- tissues.

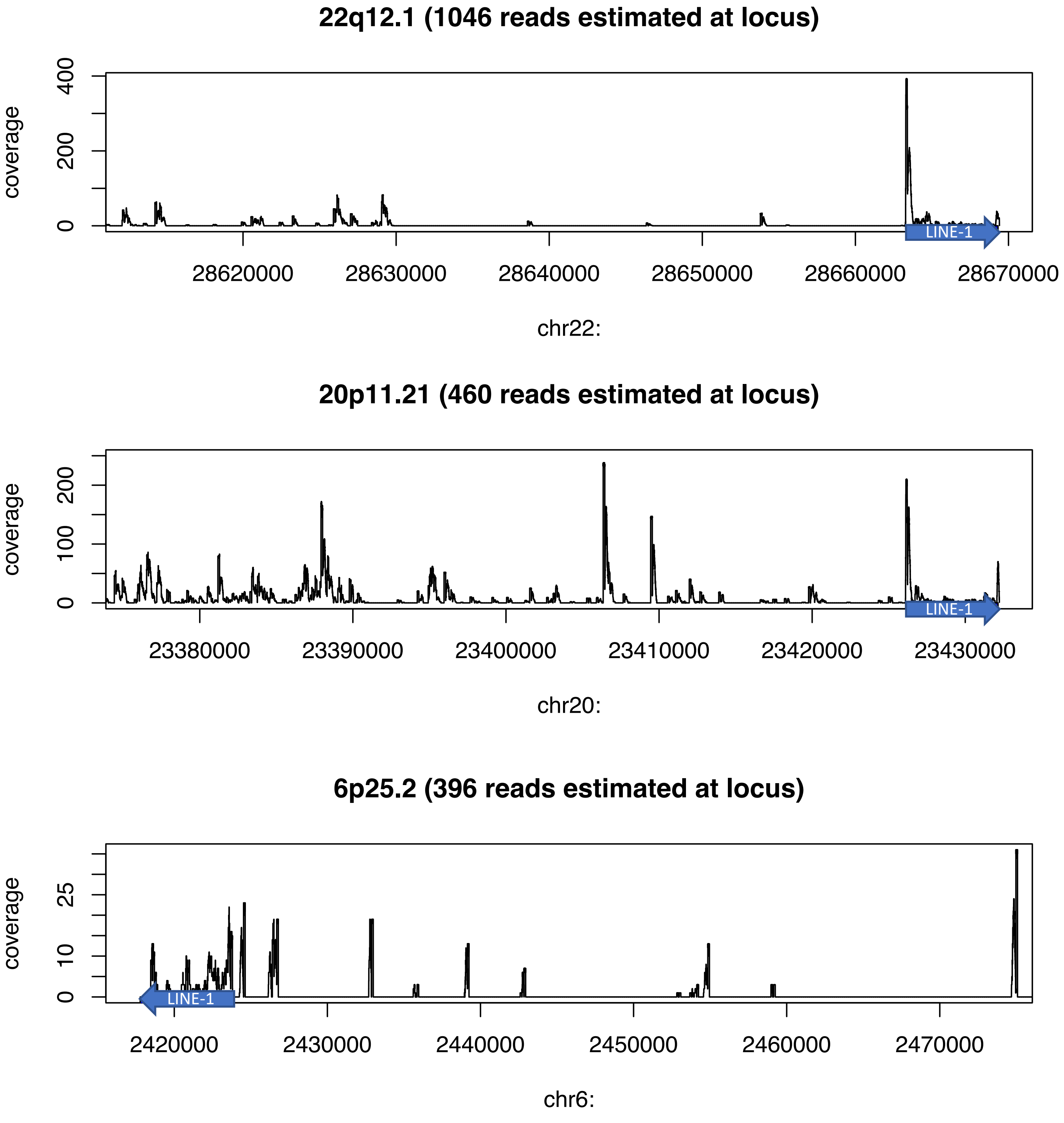

Figure S2. Coverage of reads 50kb upstream of the 3 LINE-1 loci predicted to be most highly expressed in the esophageal tissue sample based on the modified L1EM analysis. The LINE-1 locus itself is drawn in blue. On read pairs from the same strand as the element as included.

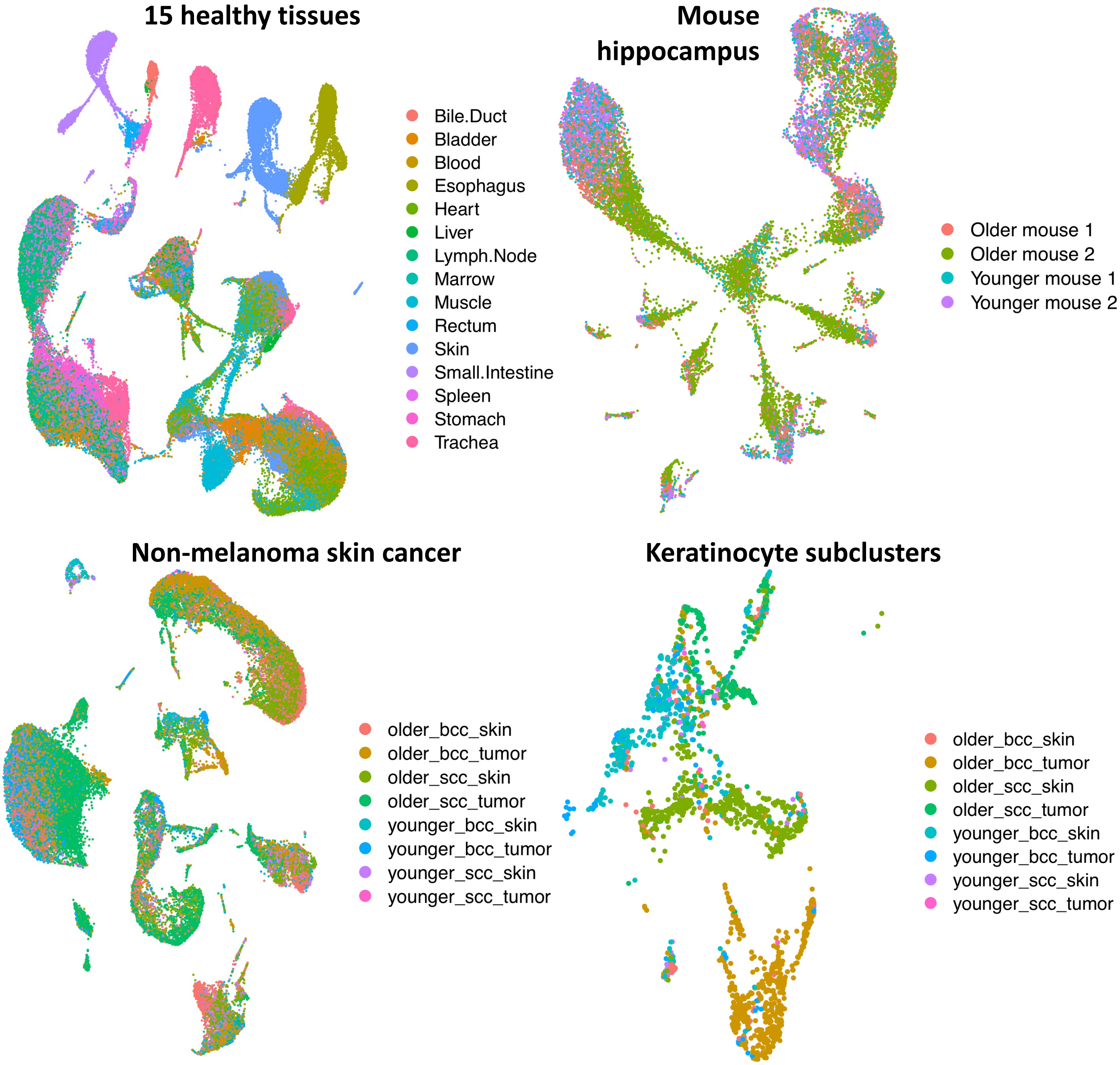

Figure S3: UMAPs colored by sample of origin.

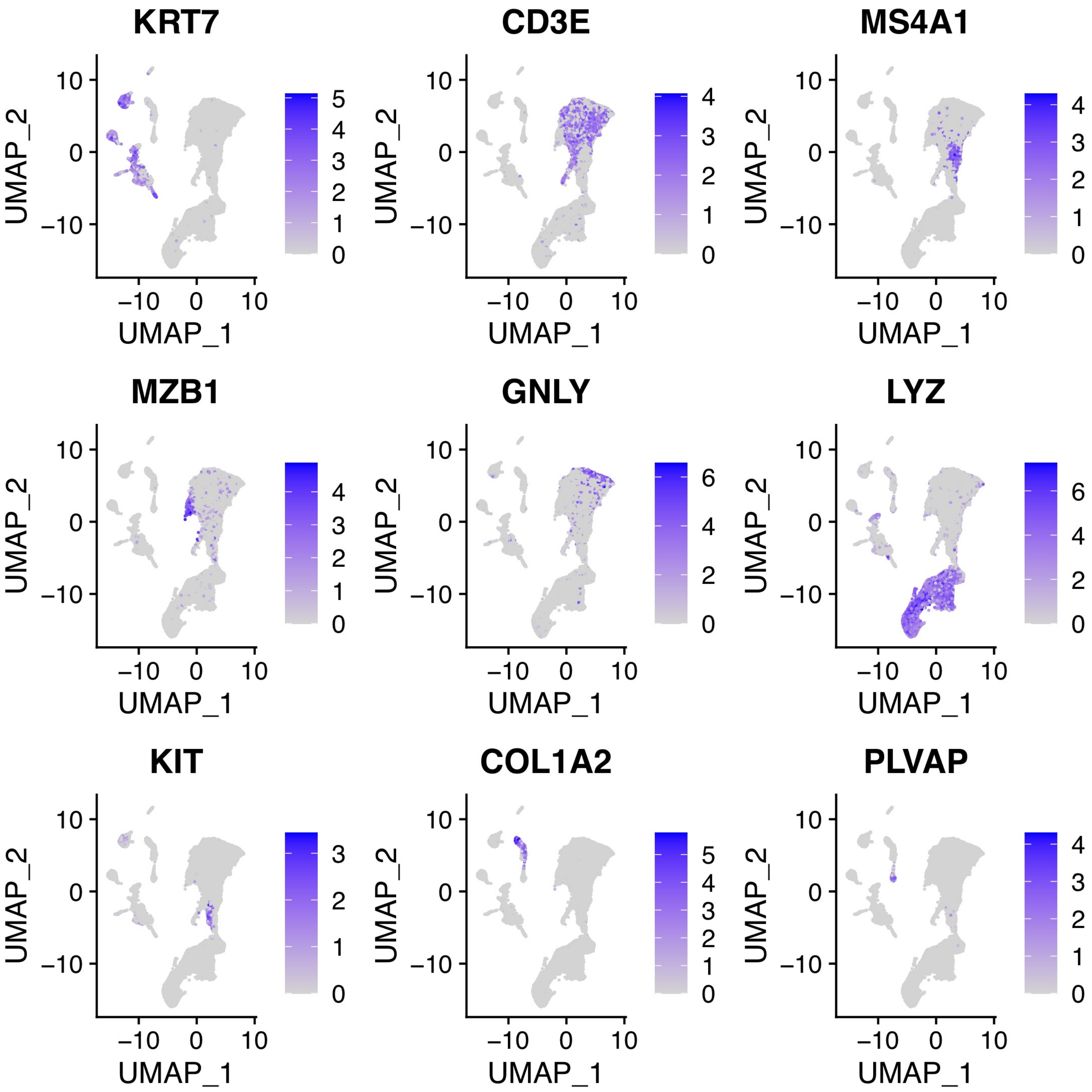

Figure S4: Marker gene expression for lung adenocarcinoma tumor, metastasis and normal cells. KRT7 = epithelial/cancer, CD3E = T cells, MS4A1 = B cells, MZB1 = plasma cells, GNLY = NK cells, LYZ = macrophages, KIT = mast cells, COL1A2 = fibroblasts, PLVAP = endothelial cells.

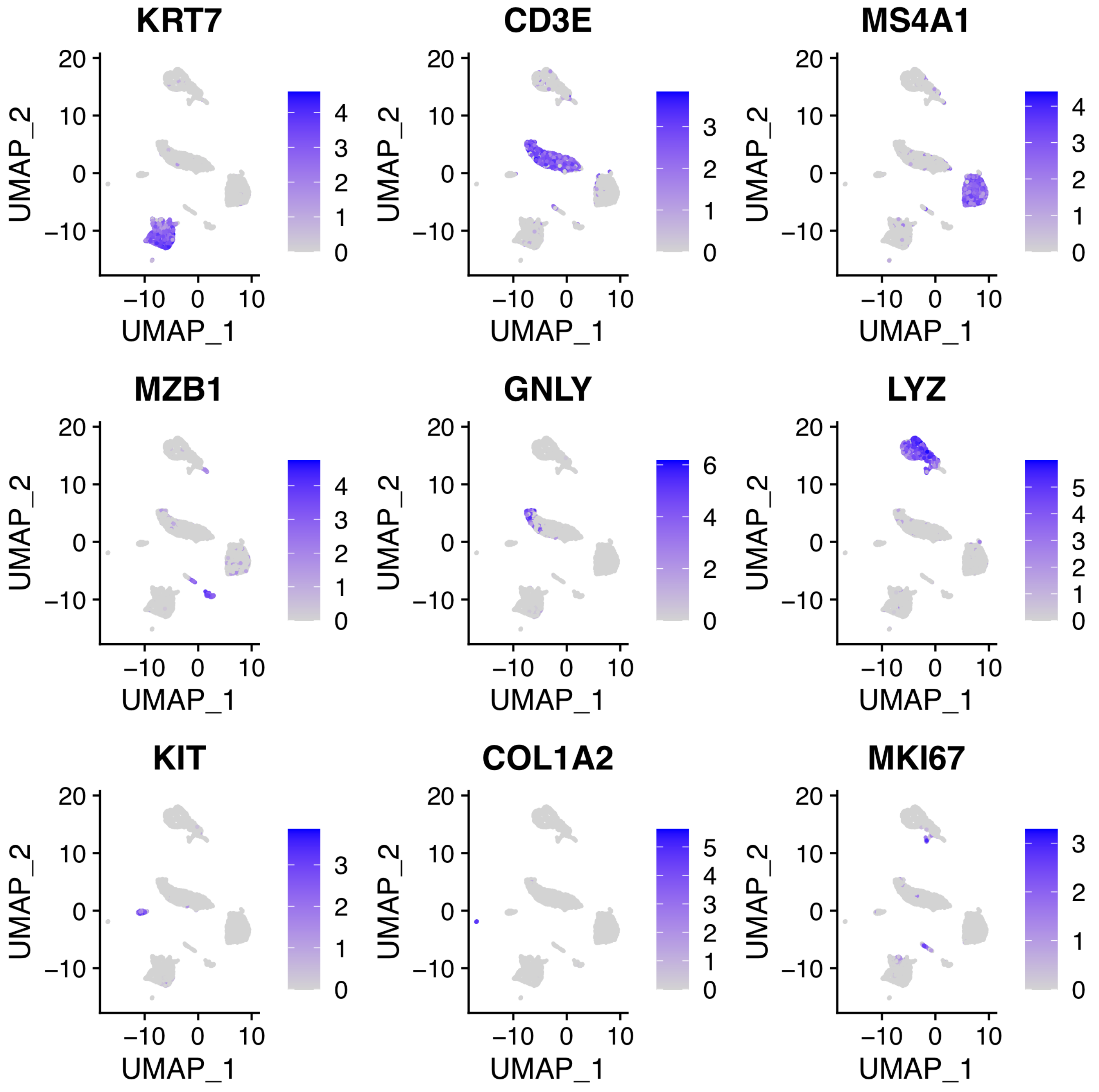

Figure S5: Marker gene expression for lung squamous cell cancer tumor cells. KRT7 = epithelial/cancer, CD3E = T cells, MS4A1 = B cells, MZB1 = plasma cells, GNLY = NK cells, LYZ = macrophages, KIT = mast cells, COL1A2 = fibroblasts, MKI67 = mitotic cells.

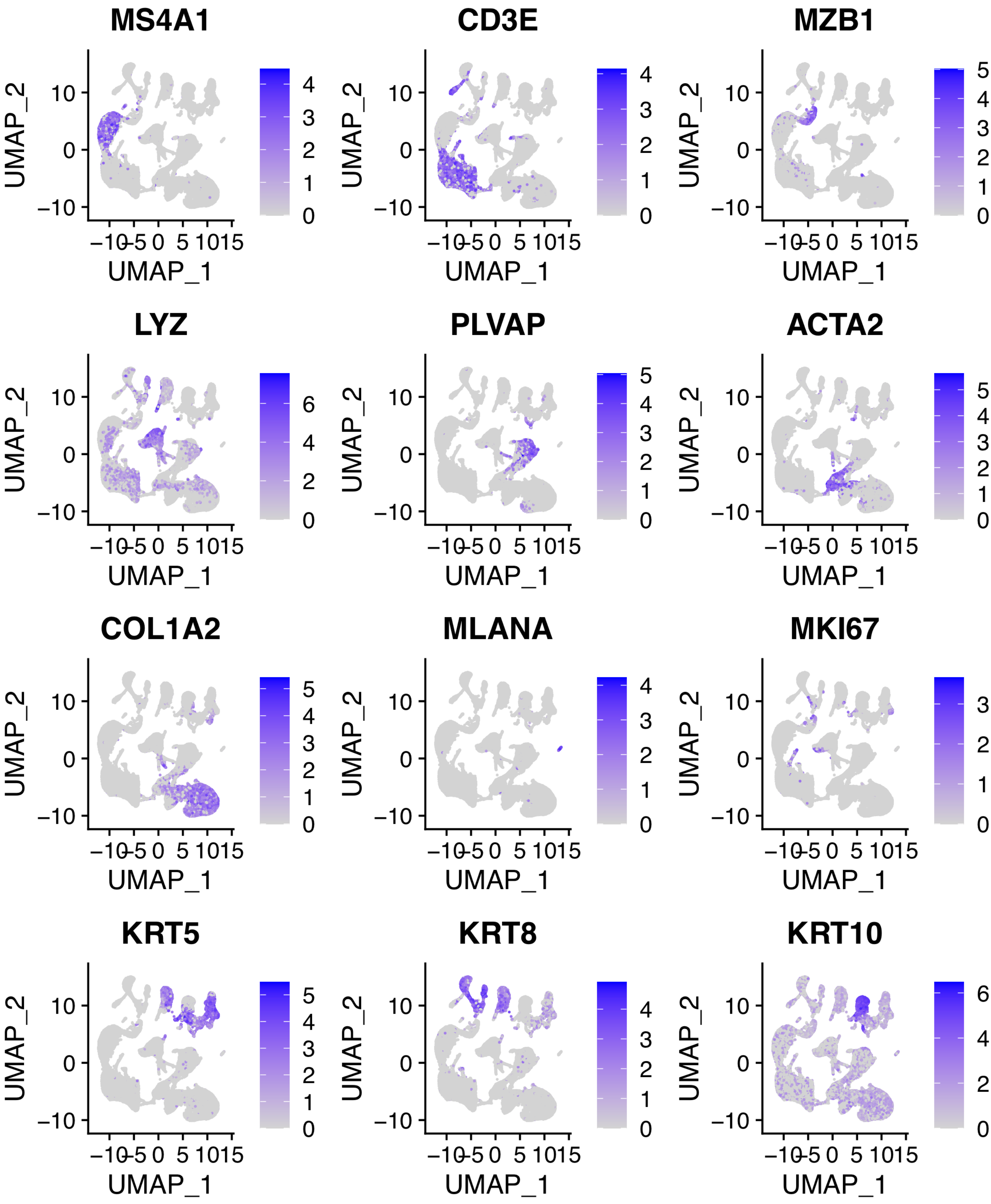

Figure S6: Marker gene expression for normal tissue cells. MS4A1 = B cells, CD3E = T cells, MZB1 = plasma cells, LYZ = macrophages, PLVAP = endothelial, ACTA2 = muscle, COL1A2 = Fibroblasts, MLANA = melanocytes, MKI67 = mitotic, KRTX = epithelial.

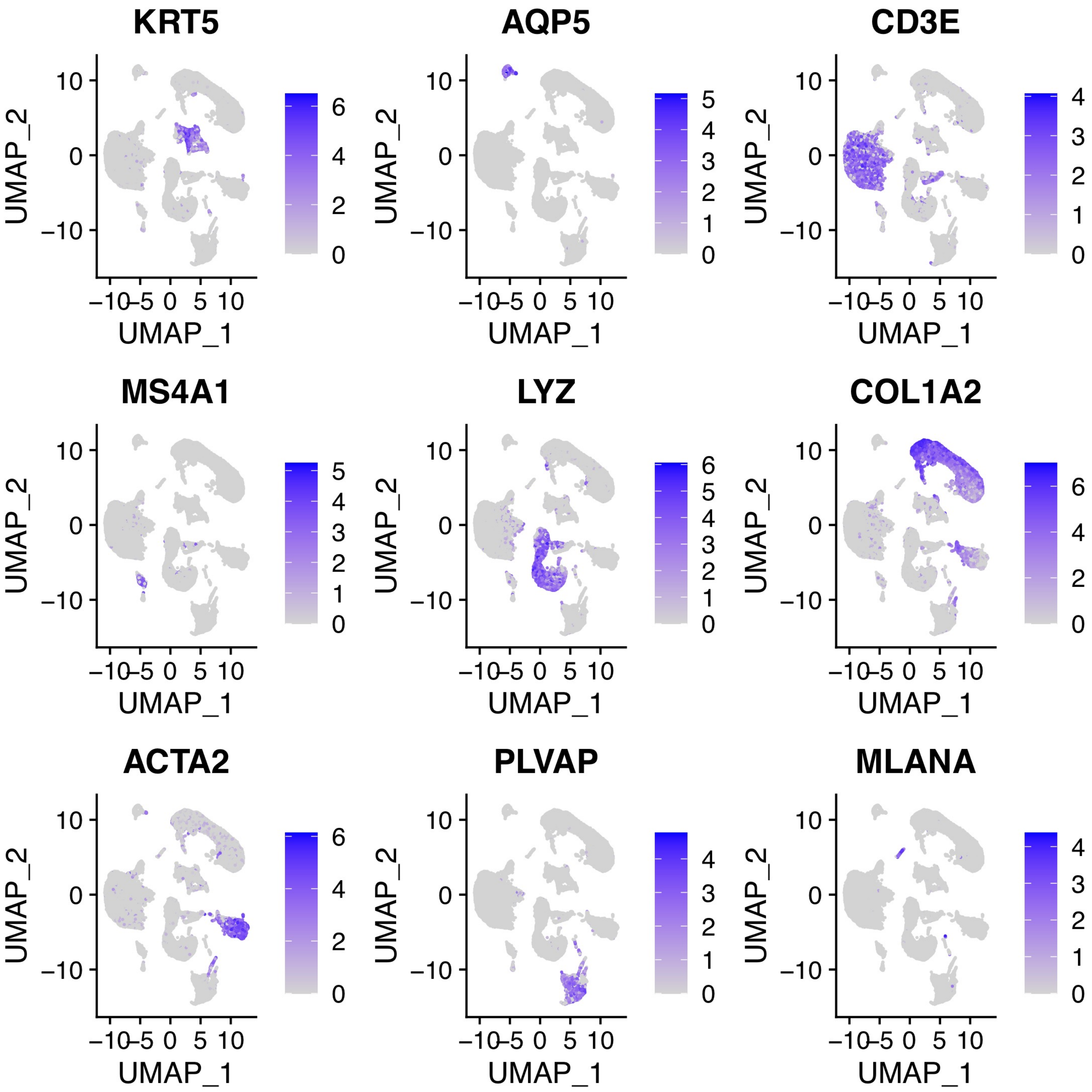

Figure S7: Marker gene expression for non-melanoma skin cancer patients.

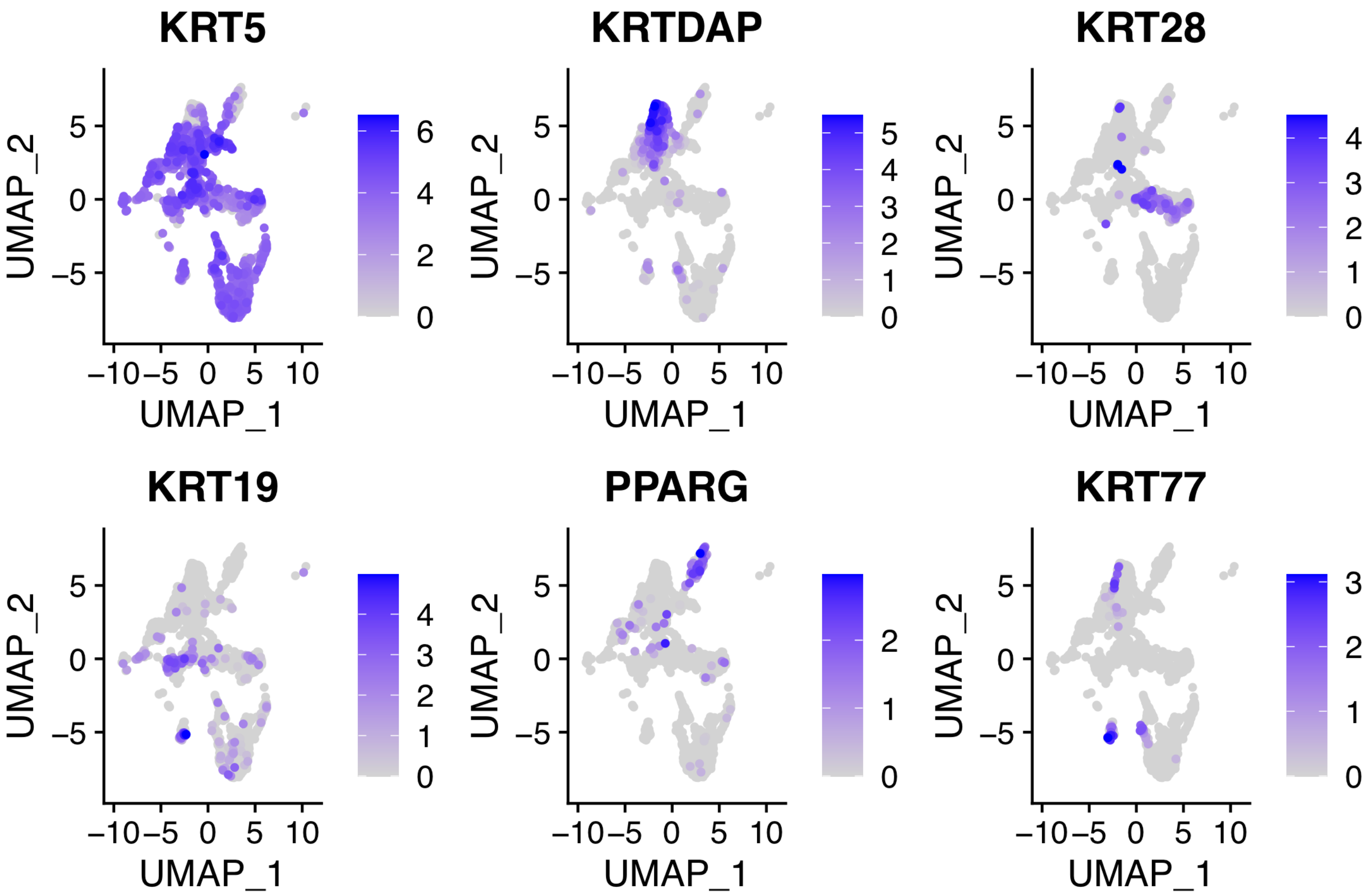

Figure S8: Marker gene expression for non-melanoma skin cancer patients.

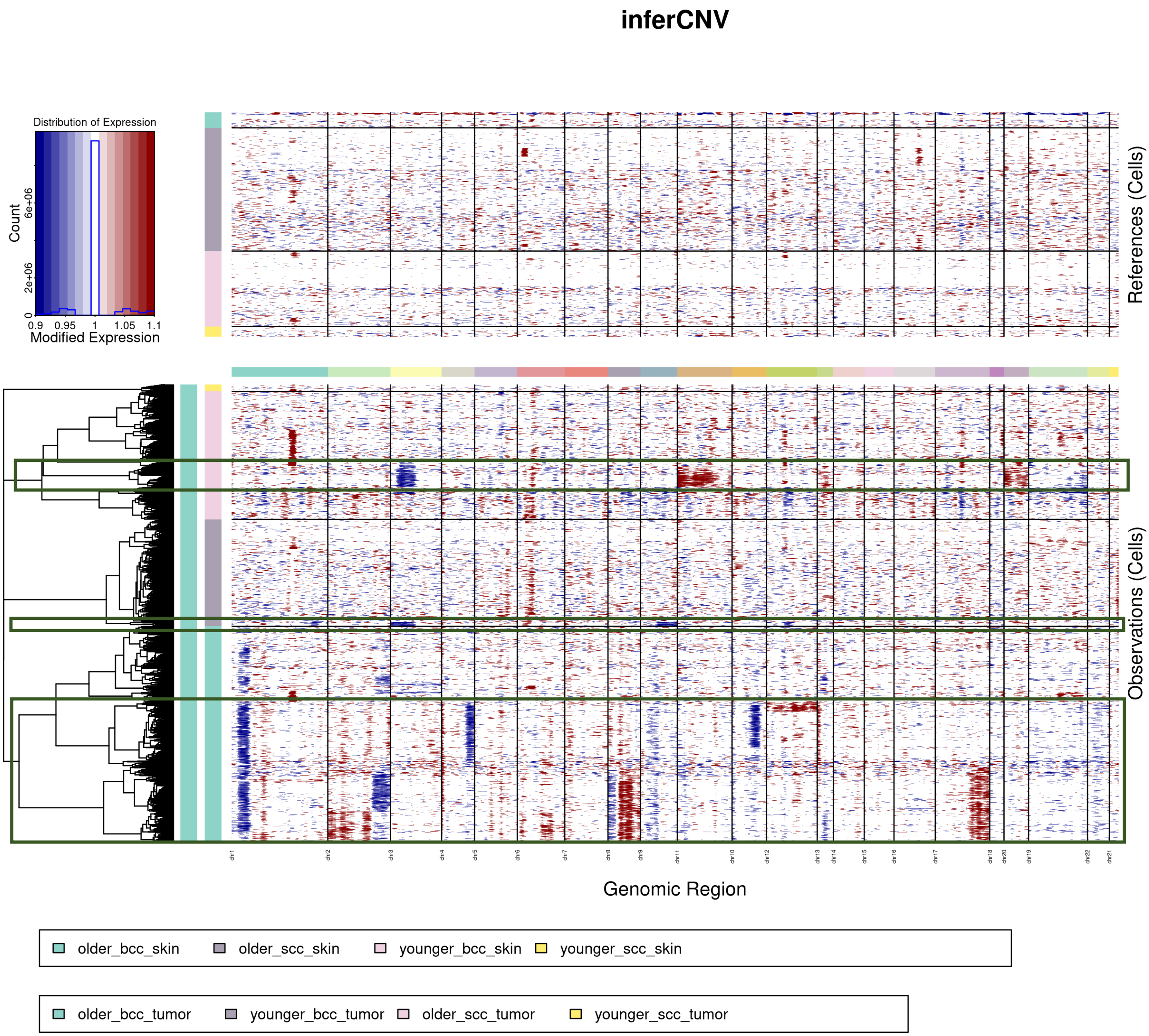

Figure S9: Output of inferCNV. CNV+ cells are highlighted by green boxes.

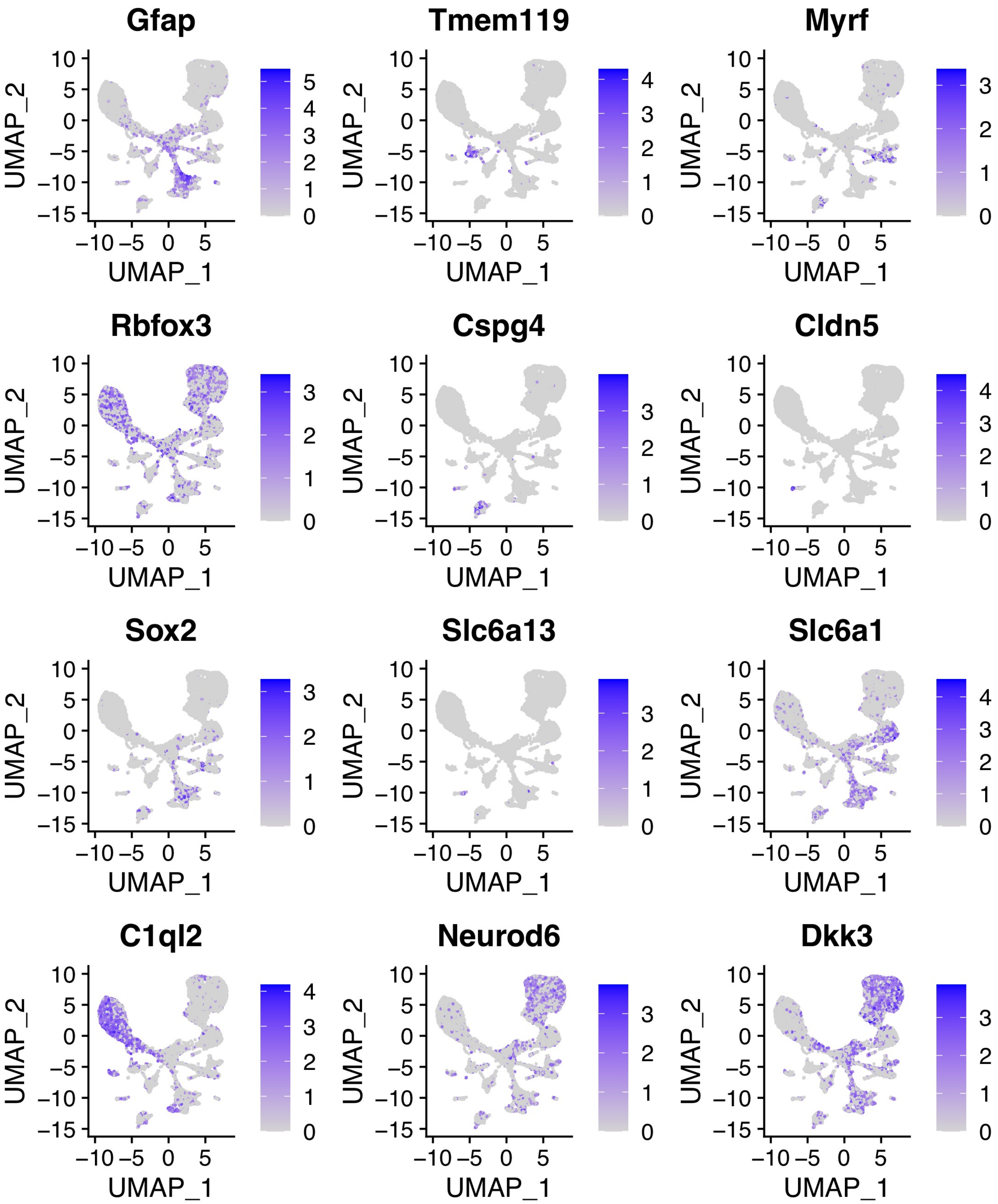

Figure S10: Marker gene expression for mouse hippocampal cells. Gfap = astrocytes, Tmem119 = microglia, Myrf = oligodendrocytes, Rbfox3 = neurons, Cspg4 = OPCs, Cldn5 = ependymal, Slc6a13 = vascular, Slc6a1 = interneurons, c1ql2 = DG neurons, Neurod6/Dkk3 = CA neurons.

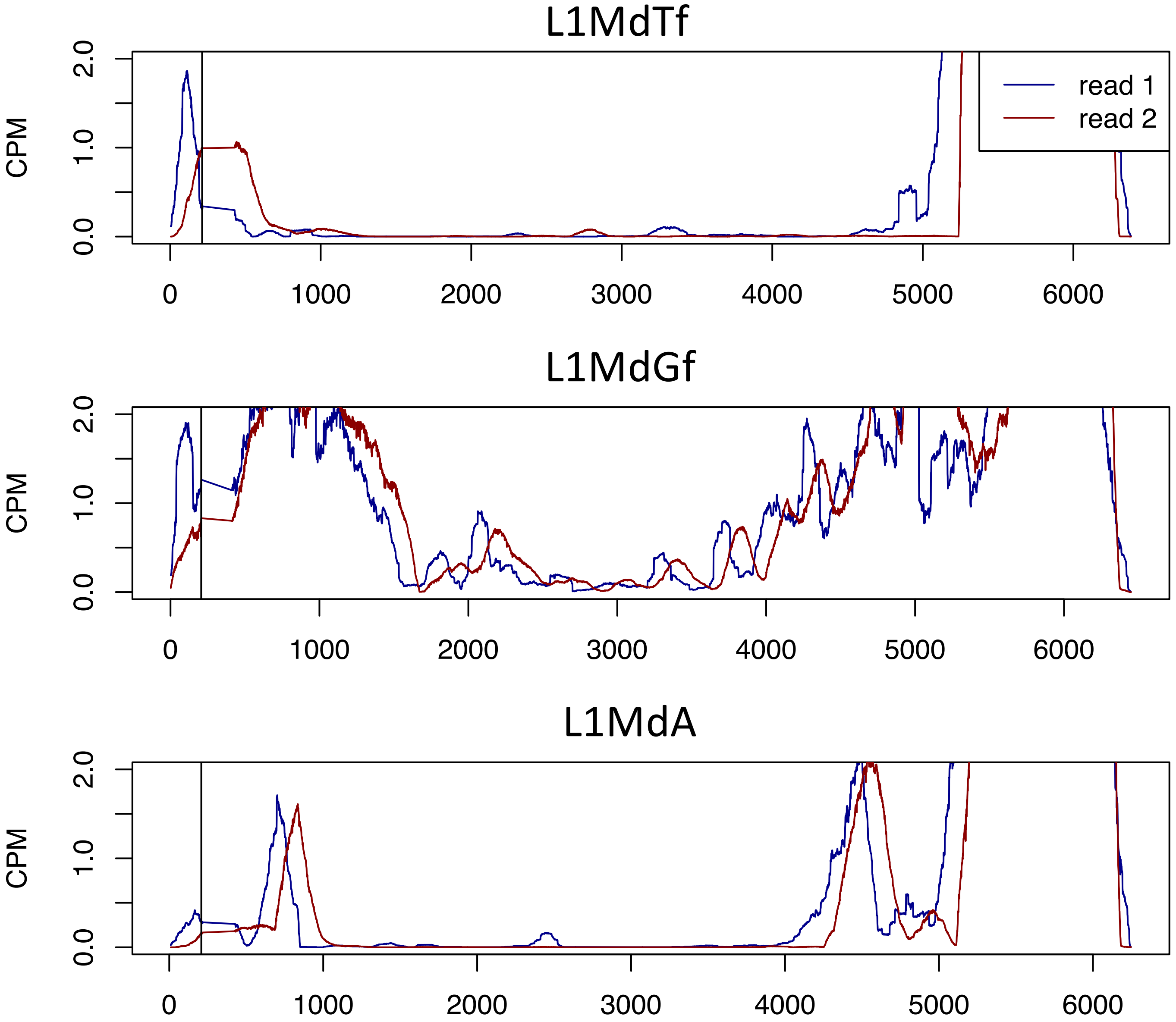

Figure S11: Read 1 and read 2 coverage across the active mouse LINE-1 consensus sequences. Read 1 must be downstream of the vertical black line to be counted toward LINE-1 expression.
