# Supplementary material for: LINE-1 retrotransposon expression in cancerous, epithelial and neuronal cells revealed by 5′ single-cell RNA-Seq
Source: Nucleic Acids Res. 2023 Feb 6;51(5):2033–45. doi: 10.1093/nar/gkad049 (PMC10018344; doi:10.1093/nar/gkad049)
Supplement: gkad049_Supplemental_Files [file gkad049_supplemental_files.zip › scLINE1_tableS1_caption.pdf]

Supplementary table 1. Datasets analyzed in this study. Information provided includes organism (human or mouse), tissue or cell line of origin, disease (cancer type if relevant and normal otherwise), age, number of cell included in our analysis, the mean number of RNA molecules detected per cell, the read lengths sequenced, and the SRA accession number. (A url is provided for the 10x genomics sample data instead as that data is not available through SRA.)
